# A case report of primary prostate intravascular large B cell lymphoma presenting as prostatic hyperplasia

**DOI:** 10.1097/MD.0000000000018384

**Published:** 2019-12-16

**Authors:** Fang Zhu, Huaxiong Pan, Yin Xiao, Qiuhui Li, Tao Liu, Xinxiu Liu, Gang Wu, Juan Li, Liling Zhang

**Affiliations:** a Cancer Center; b Department of Pathology, Union Hospital, Tongji Medical College, Huazhong University of Science and Technology, Wuhan, China.

**Keywords:** immunochemotherapy, intravascular large B cell lymphoma, prostate, prostatic hyperplasia

## Abstract

**Rational:**

Intravascular large B-cell lymphoma (IVLBCL) is a rare condition with a poor prognosis. The clinical presentation of primary lymphoma of the prostate is non-specific and it is difficult to distinguish from other prostatic diseases. The primary prostate IVLBCL is very rare, the diagnosis and treatment of which remains unclear. We reported a rare case to explore the diagnosis and treatment for the primary prostate IVLBCL.

**Patients concerns:**

This report described a case of a 71-year-old male diagnosed as primary prostate IVLBCL who presented with prostatic hyperplasia.

**Diagnosis:**

The patient first visited an outpatient clinic of urinary surgery because of urinary urgency and frequency and was diagnosed as benign prostatic hyperplasia in about January 2010. Four years later, the symptoms worsened quickly within two months. The diagnosis was still prostatic hyperplasia according to the physical examination and imaging. However, histopathology showed IVLBCL of prostate after transurethral resection of the prostate.

**Interventions:**

With the clear diagnosis of primary prostate stage I IVLBCL, the patient received immunochemotherapy of R-CHOP (rituximab, cyclophosphamide, adriamycin, vincristine, and prednisolone) for 4 cycles and intensity-modulated radiation therapy (IMRT) including the region of prostate with the dose of 45Gy/25f.

**Outcomes:**

The response was complete remission after all treatment. The last follow-up time of the patient was June 20th, 2019, and no evidence of disease progression was observed. The progression-free survival of the patient was about 49 months until now.

**Lessons:**

The biopsy of prostate by prostatectomy plays an important role in the diagnosis and removal of the original lesion of primary prostate lymphoma. There is no consensus on therapeutic modalities for the treatment of primary prostate IVLBCL till now. Individual treatments include immunochemotherapy and/or radiotherapy according to the National Comprehensive Cancer Network (NCCN) practice guideline of diffuse large B cell lymphoma (DLBCL) based on the performance status and tumor staging of the patient. Timely and accurate diagnosis as well as the appropriate treatment may improve the clinical outcome.

## 1 Introduction

IVLBCL is a rare subtype of extranodal DLBCL with poor prognosis, which is highly aggressive and may involve any tissue and organs.^[1–4]^ Central nervous system,^[5]^ bone marrow^[6]^ and skin^[7]^ are the most common systems involved with IVLBCL. It is different from the majority types of lymphoma presenting as the enlargement of lymph nodes. The histopathology of IVLBCL is characterized by the proliferation of neoplastic cells within the vascular lumen, especially within capillaries. So, the diagnosis of IVLBCL is often difficult because of the absence of specific clinical presentation, laboratory and imaging findings. Primary lymphoma of prostate is a rare condition that only accounts for 0.09% of all prostate neoplasms and 0.1% of all non-Hodgkin's lymphoma (NHL).^[8]^ The prostate has been reported as one of the involved sites by IVLBCL in few cases.^[9–11]^ However, primary prostate IVLBCL was reported only in three cases according to a comprehensive literature search from the electronic databases PubMed with the keywords of “intravascular large B cell lymphoma” and “prostate”.^[12–14]^ It is lack of the accurate diagnosis and treatment strategy of primary prostate IVLBCL until now. Here we report a case of long-term survival with primary prostate IVLBCL in a 71-year-old male who presented with prostatic hyperplasia.

## 2 Case report

**Figure 1:**
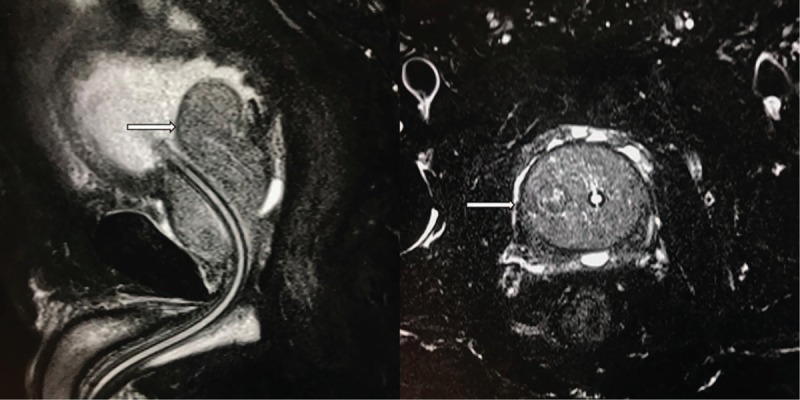
The MRI scan of the prostate showed the enlarged prostate gland (arrow) in T2 enhanced-weighted imaging.

**Figure 2:**
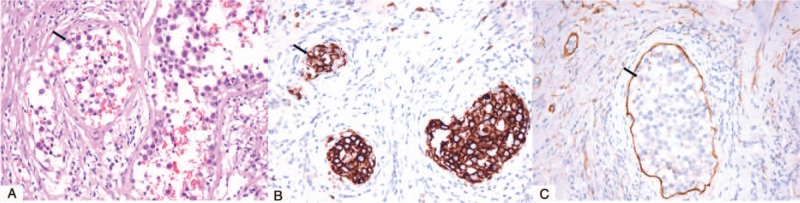
Pathology of intravascular B cell lymphoma of the prostate. H&E staining showed atypical large lymphoid cells(arrow) within the lumina of small and intermediate-sized blood vessels. (original magnification ×400). Immunohistochemistry staining showed that the tumor cells(arrow) were CD20-positive confirming the presence of B lymphocytes. (original magnification ×400). CD34 immunohistochemistry staining highlighted the intravascular growth pattern of tumor cells(arrow). (original magnification × 400).

A 71-year-old male first visited an outpatient clinic of urinary surgery because of urinary urgency and frequency and was diagnosed as benign prostatic hyperplasia in about January 2010. He was treated with Tamsulosin which was an α-blocker and the symptoms relieved after taking the medicine. But the symptoms worsened quickly within 2 months since September 2014. He also had hematuria occasionally during the past 2 months without fever, night sweats, weight loss or any other preceding symptoms. He visited an outpatient clinic of urinary surgery again in November 2014. Physical examination was normal. Ultrasound examination showed enlarged prostate without nodular surface which was measured as 8 × 4 × 6 cm^3^. Magnetic resonance imaging (MRI) scan of the prostate showed the enlarged prostate without nodular surface in T2 enhanced weighted imaging (Fig. 1). His complete blood count, lactate dehydrogenase (LDH) and serum prostate-specific antigen (PSA) level were normal. He had no personal or family medical history of malignant neoplasm and urinary system infection. The diagnosis was still prostatic hyperplasia according to physical examination and imaging. Transurethral resection of the prostate (TURP) was performed in November 2014. The symptoms regressed after the operation. Astonishingly, the histological, and immunohistochemical studies of the prostatectomy showed an atypical, intravascular population of cells, not prostatic hyperplasia. The cells were large and featured. The atypical population showed positive staining for CD20, CD10, multiple myeloma oncogene 1(MUM-1), Bcl-6, CD34, negative staining for CD3, ALK, CK, CD30, CyclinD1, and exhibited a high proliferation index as illustrated by Ki-67 staining (98% positive) (Fig. 2). The features were consistent with IVLBCL. The patient was diagnosed with IVLBCL in the prostate. He went to the department of lymphoma to continue the specialized therapy. A further ^18^FDG positron emission tomography-computed tomography (PET-CT) scan of the whole body showed no significantly increased metabolic activity in the region of the prostate gland. No other areas of increased metabolic activity were observed, either. Bone marrow biopsy showed no evidence of lymphoma infiltration. The final diagnosis of this patient was primary prostate IVLBCL, germinal center B-cell subtype, stage IA according to the Ann Arbor staging criteria, and International Prognostic Index (IPI) score was 1. The patient was classified as the low-risk group based on the IPI score. He received 4 courses of immunochemotherapy with R-CHOP. Followed by 2 courses and 4 courses of chemotherapy respectively, the repeated computed tomography (CT) scan from neck to pelvic cavity revealed that the response to the therapy was complete remission. He received intensity-modulated radiation therapy (IMRT) of the prostate after chemotherapy, and the dose of the clinical target volume (CTV) including the region of prostate was 45Gy/25f. He finished all treatment in May 2015 and is still alive now with a good quality of life.

## 3 Discussion

IVLBCL is a rare subtype of NHL with a poor prognosis. IVLBCL was first described in 1959 by Pfleger and Tappeiner^[15]^ and was characterized by the diffuse proliferation of malignant B-cells in small- and medium-size vessels, especially in capillary and postcapillary venule. It can involve any organ of the body such as kidney,^[16]^ spleen, skin,^[17]^ uterus, ovaries, central nervous system, bone marrow, and so on. Skin, central nervous system, and bone marrow are the most common systems involved. The clinical presentation of IVLBCL is non-specific, which is associated with the involved organ, and it is different from the enlargement of the lymph node that is most of the presentation of lymphoma. Only few cases were reported until now because of the low morbidity and difficulty in diagnosis. Many cases were diagnosed at autopsy. The World Health Organization (WHO) classified it as a specific, rare subtype of NHL in 2016^[18]^ with an estimated annual incidence of fewer than 0.5/1,000,000.^[19]^ Given the high variance of clinical presentation, many diagnostic methods failed to show all involved organs exactly. Then, the PET/CT scan of the whole body plays an important role in the diagnosis, staging, and evaluation of treatment response in IVLBCL, in which all involved tissues are showed increased metabolic ^18^F-fluorodeoxyglucose activity significantly.^[20,21]^

Prostate cancer is the most common malignant tumor among the elderly men worldwide, but primary lymphoma of the prostate is a rare condition that only accounts for about 0.09% of all prostate malignancies, representing about 0.2% to 0.8% of extranodal lymphomas.^[22]^ Many reported cases of prostate involvement by NHL accompanied with other lymph nodes or organs involved, which represented the late manifestation of advanced lymphoma. It is liable to be neglected owing to the lack of specific findings in the early stage of the prostate involved. It is usually detected by the CT scan or PET/CT scan from head to pelvic cavity when the patients with lymphoma received the staging examination. The symptom of lymphoma involved in the prostate is untypical. Urinary urgency and frequency are the most common presentation because of the enlarged prostate gland. Therefore, it is easy to misdiagnose prostate neoplasms and benign prostatic hyperplasia. Biopsy of the prostate is the only way to confirm the diagnosis of lymphoma involvement. Various subtypes of NHL of the prostate have been reported including DLBCL, follicular lymphoma,^[23]^ Burkitt lymphoma, mantle cell lymphoma, mucosa-associated lymphoid tissue lymphoma,^[24]^ and so on. DLBCL remains the most common subtype of prostate lymphoma. Primary NHL of the prostate is characterized by the presence of an enlarged prostate at the beginning of the disease, localization of lymphoma to the prostate gland, and the absence of involvement of any other organ or lymph node within 1 month of diagnosis according to the study of Bostwick and Mann.^[25]^ Primary prostate IVLBCL is rare, and the presentation of which is the same as the primary NHL of the prostate. Therefore, it is easy to confuse with prostatic hyperplasia.

Currently, there is no consensus on therapeutic modalities for the treatment of primary prostate IVLBCL because of the small number of cases. Systemic chemotherapy is necessary because IVLBCL is identified highly aggressive. Ponzoni et al^[2]^ reported that anthracycline-based chemotherapy was associated with nearly a 60% response rate and a 3-year overall survival rate higher than 30% in the patients with IVLBCL, which was better than those of non-anthracycline-based chemotherapy. CHOP or CHOP-like regimens have been reported to be effective in some IVLBCL cases.^[13]^ Shimada reported that the addition of rituximab to CHOP or CHOP-like regimen revealed a better complete response rate, overall survival and 2-year progression-free survival than those of chemotherapy alone in the patients with IVLBCL.^[26]^ Despite the lack of standard therapy based on a large sample of clinical trials, the chemotherapy of R-CHOP with or without radiotherapy remains the currently recommended therapy for IVLBCL according to the available data^[26,27]^ and NCCN practice guideline of DLBCL. The patient was diagnosed as primary prostate IVLBCL stage I according to the Ann Arbor staging criteria and was classified as the low-risk group based on the IPI score. The patient received 4 courses of chemotherapy of R-CHOP and IMRT including the area of the prostate with the dose of 45Gy to improve the local control according to the NCCN guideline of DLBCL. Although the reported prognosis of IVLBCL is very poor, the patient has survived for 4 years without disease progression until now. Therefore, timely and accurate diagnosis as well as the appropriate treatment may improve the clinical outcome.

In conclusion, primary IVLBCL of the prostate is rare and clinically difficult to distinguish from benign prostatic hyperplasia and prostatic carcinoma as it occurs in the same age group of 6th decade and presents with the similar obstructive urinary symptoms. Thus, the biopsy of the prostate by prostatectomy is very important in the diagnosis and removal of the original lesion of primary prostate lymphoma. The PET-CT is useful for the diagnosis, staging, and assessment of the response of IVBCL.R-CHOP may be an effective chemotherapy regimen to primary prostate IVLBCL as the first-line treatment. Radiation may improve the local control to the patient with primary prostate lymphoma at the early stage. Earlier diagnosis and appropriate treatment may improve the patient's survival time and life quality.

## Acknowledgments

We thank the patient and his families as well as all doctors in the department of pathology and Cancer Center, Union Hospital, Tongji Medical College, Huazhong University of Science and Technology.

## Author contributions

**Data curation:** Fang Zhu, Huaxiong Pan, Yin Xiao, Qiuhui Li, Tao Liu, Xinxiu Liu, Gang Wu, Juan Li, Liling Zhang.

**Funding acquisition:** Liling Zhang.

**Methodology:** Huaxiong Pan, Gang Wu.

**Supervision:** Gang Wu, Juan Li.

**Validation:** Fang Zhu, Huaxiong Pan, Juan Li, Liling Zhang.

**Writing – original draft:** Fang Zhu.

**Writing – review & editing:** Liling Zhang.
